# Wall Tension and Tubular Resistance in Kidney Cystic Conditions

**DOI:** 10.3390/biomedicines11061750

**Published:** 2023-06-18

**Authors:** Michele Della Corte, Davide Viggiano

**Affiliations:** Department of Translational Medical Sciences, University of Campania Luigi Vanvitelli, 80131 Naples, Italy; micheledellacorte@outlook.it

**Keywords:** ADPKD, Laplace’s law, cysts, kidney disease

## Abstract

The progressive formation of single or multiple cysts accompanies several renal diseases. Specifically, (i) genetic forms, such as adult dominant polycystic kidney disease (ADPKD), and (ii) acquired cystic kidney disease (ACKD) are probably the most frequent forms of cystic diseases. Adult dominant polycystic kidney disease (ADPKD) is a genetic disorder characterized by multiple kidney cysts and systemic alterations. The genes responsible for the condition are known, and a large amount of literature focuses on the molecular description of the mechanism. The present manuscript shows that a multiscale approach that considers supramolecular physical phenomena captures the characteristics of both ADPKD and acquired cystic kidney disease (ACKD) from the pathogenetic and therapeutical point of view, potentially suggesting future treatments. We first review the hypothesis of cystogenesis in ADPKD and then focus on ACKD, showing that they share essential pathogenetic features, which can be explained by a localized obstruction of a tubule and/or an alteration of the tubular wall tension. The consequent tubular aneurysms (cysts) follow Laplace’s law. Reviewing the public databases, we show that ADPKD genes are widely expressed in various organs, and these proteins interact with the extracellular matrix, thus potentially modifying wall tension. At the kidney and liver level, the authors suggest that altered cell polarity/secretion/proliferation produce tubular regions of high resistance to the urine/bile flow. The increased intratubular pressure upstream increases the difference between the inside (Pi) and the outside (Pe) of the tubules (∆P) and is counterbalanced by lower wall tension by a factor depending on the radius. The latter is a function of tubule length. In adult dominant polycystic kidney disease (ADPKD), a minimal reduction in the wall tension may lead to a dilatation in the tubular segments along the nephron over the years. The initial increase in the tubule radius would then facilitate the progressive expansion of the cysts. In this regard, tubular cell proliferation may be, at least partially, a consequence of the progressive cysts’ expansion. This theory is discussed in view of other diseases with reduced wall tension and with cysts and the therapeutic effects of vaptans, somatostatin, SGLT2 inhibitors, and potentially other therapeutic targets.

## 1. Introduction

In 1586, a surgeon reported for the first time the presence of multiple cysts in a kidney while proceeding with the mummification of the King of Poland, Stephen Bathory [1]. Up to the 1950′s, isolated renal cysts were classified as a specific form of tumor [2].

Since then, we know that single or multiple cysts accompany several renal diseases. We distinguish (i) genetic forms, of which we will focus on adult dominant polycystic kidney disease (ADPKD) that is the most frequent hereditary form, and (ii) acquired cystic kidney disease (ACKD).

We will first review the hypothesis of cystogenesis in ADPKD and then focus on ARCD, showing that they share essential pathogenetic features, which can be explained by a localized obstruction of a tubule and/or an alteration of the tubular wall tension.

### ADPKD Cystogenesis

Adult dominant polycystic kidney disease (ADPKD) is a genetic disease characterized by the development of multiple renal cysts with progressive loss of renal parenchyma [3,4,5,6].

Forty years ago, Cuppage et al. discussed, regarding ADPKD, “whether the tubules enlarge initially because of increased intratubular pressure, as a consequence of a highly compliant basement membrane, or a combination of these factors” [7].

In 1983, Grantham also discussed the possibility that cystogenesis is due to “obstruction of tubule fluid flow by hyperplastic tubule cells, increased compliance of the tubule basement membranes and increased radial growth of cells”. However, in 1987, direct measure in experimental models reported a failure to show changes in the viscoelastic mechanical properties of basement membranes in cystic conditions [8]. However, as discussed below, a review of the data reported in this study showed an increased deformability of tubules in animal models with cysts compared to the controls.

Unfortunately, the hypothesis of cystogenesis in ADPKD as driven by an increase in intratubular pressure or a highly compliant basement membrane has been abandoned in favor of other theories insisting on cell proliferation or fluid secretion. Indeed, the physical properties of the kidneys, such as intratubular pressure and basement membrane compliance, are today almost entirely forgotten in the modern era of vapatans. Here, we will briefly review the main theories on cystogenesis and their limitations and propose a different interpretation of the available data.

The current theories on cystogenesis in ADPKD are the following:

- Increased fluid secretion from tubular cells. It has been observed that the cyst fluid from patients with ADPKD has effects on cell secretion in vitro [9]. This led to the hypothesis that fluid secretion was important for cyst formation. Furthermore, two subjects with both cystic fibrosis (which should induce altered tubular secretion) and ADPKD were noted to have a milder kidney phenotype. This observation was difficult to confirm [10]. The hypothesis was that the anomalous transmembrane conductance regulator (CFTR) in cystic fibrosis could modulate cyst expansion. Within the same direction, another hypothesis suggests that epithelial cells in ADPKD have altered cyclic AMP, and this increases secretion and, therefore, cyst formation. Overall, this secretion hypothesis does not explain the extrarenal “signatures” of ADPKD (see below).
- Epithelial cell proliferation. Cyst fluid from patients with ADPKD does not only change cell secretion but also cell proliferation in vitro [9]. It is unclear if this also happens in humans and in vivo. However, the research has focused on possible mediators of cell proliferation, such as mammalian target of rapamycin (mTOR), tuberin [11], a shift to aerobic glycolysis [12,13], JAK-STAT, IL-13, STAT6 [14], EGFR/EGF, and TGFa [15]. ERK is also involved in cyst expansion and cellular proliferation by its association with the scaffolding protein PEA15 [16,17]. The major problem of this theory is that cell proliferation is more likely an effect of cyst enlargement rather that its cause, as discussed below. Furthermore, it does not explain the extrarenal “signatures” of ADPKD (see below).

Both increased fluid secretion and epithelial cell proliferation are hypothesized to be linked to the genetic alteration of the ADPKD genes through a dysfunction of the primary cilium. The premise of this hypothesis is that the products of the PKD1 and PKD2 genes linked to ADPKD colocalize with the primary cilium [18,19] in the embryonic kidney and in cell cultures of collecting ducts (cells grown in proliferation media never show this property). These proteins, when paired with the primary cilium, appear to regulate calcium signaling following fluid-flow stress on the cell surface. Indeed, calcium-binding proteins, such as calcium also Ca(2+)/calmodulin-dependent protein kinase II (CaMKII), might be involved in cyst growth [20,21], behavioral alterations, and changes in Ca/calmodulin kinase II levels in the striatum of connexin36-deficient mice. Therefore, the genetic alteration of PKD1/PKD2 decreases the ability of the collecting duct cells to sense the direction of intratubular fluid flow. This would disrupt the normal orientation of the cells (planar polarity), thus modifying their geometry. In experimental models, this was demonstrated in embryonic tissues with cysts already at birth [22] or in Drosophila or in genetic models unrelated to ADPKD [23]. The hypothesis states that the abnormal ciliary function/cell polarity then produces cysts because of cell division occurring in the wrong direction and altered fluid secretion (discussed above). This hypothesis has the limitation that it does not take into account the extratubular and extrarenal expression of PKD1/PKD2 [24]. Indeed, the Human Protein Atlas database (https://www.proteinatlas.org/, 1 May 2023) reports that PKD1/PKD2 are expressed in proximal tubule cells, colon, skin, and other organs that do not have a primary cilium.

Overall, although these molecular hypotheses may seem attractive, they have several difficulties:

(1) If cystogenesis is due to cell proliferation, anti-proliferative drugs should be therapeutic for ADPKD. However, anti-proliferative agents, such as sirolimus and everolimus, do not stop cyst growth in ADPKD (reviewed in [25]).

The only exception is in regards to somatostatin analogues (octreotide [26] such as lanreotide [27]).The cyst-reducing effect of somatostatin (SS) analogues has been reproduced by two different somatostatin analogues and is consistent with the strong expression of somatostatin receptor type 2 in distal tubules according to the Human Protein Atlas (see also [26]). This phenomenon has been hypothesized to occur by inhibiting cell growth. However, the effects of SS on tubular cell growth rely on only one evidence published in 1996 on the opossum kidney cell line “OK” [28]. Conversely, it is well known that SS exerts potent effects on kidney water handling [26] as well as on the glomerular filtration rate [26]. Therefore, as discussed below, the effects of SS are better explained by its role on tubular/glomerular physiology rather than by anti-proliferative effects.

Cell proliferation certainly occurs in cysts simply because, as the cysts enlarge (due to other mechanisms), the tubular epithelium must grow to cover them. Cell growth is an effect and not the cause of cyst enlargement. As observed by Grantham already in 1987, cysts cannot be covered by simply stretching the tubule epithelium; new cells are required to cover them [29].

Here, we underline, however, that an altered growth/secretion/polarity in normal tubules may create regions of obstruction and high resistance to urine flow [30], thus favoring cyst formation upstream of the obstruction.

(2) If cell secretion or proliferation cause cystogenesis, the latter should be unrelated to the glomerular filtration rate (GFR). Conversely, when GFR is significantly reduced or absent (end-stage kidney disease, ESKD), no further growth of the cysts is evident [26]. The dependence of cyst formation on glomerular filtration suggests that intratubular pressure is an important factor for cystogenesis.

(3) Patients with ADPKD have aneurysms and hernias, which are unexplained by the current theories (see below).

(4) None of the above-mentioned mechanisms can explain why a similar phenotype arises from alterations of seemingly completely unrelated genes, namely PKD1, PKD2, GANAB, and DNAJB11 (see below).

(5) If ADPKD depends only on cell proliferation, we would also expect the formation of solid masses as in tumors [31], which is very rare in ADPKD.

(6) Cystogenesis also occurs in several other conditions (e.g., ageing, cyclosporine use; see last paragraph on ARCD) where altered polarity/cell growth, etc. are unlikely to be responsible for the phenomenon.

(7) Cysts also develop in other organs such as the liver. It is certain that liver cystogenesis does not involve Tolvaptan-related mechanisms as this drug has no effects on liver cysts. However, liver cystogenesis seems to respond to somatostatin analogues. Furthermore, polycystic liver disease is due to a mutation in the Prkcsh and Sec63 genes, which do not encode for proteins involved in the primary cilium.

Therefore, the ADPKD theory based on cell proliferation and fluid secretion does not explain the full phenotype of the systemic disease and may be in contrast with empirical evidence.

At variance, the original observation by Cuppage et al. regarding a possible reduction in wall tension in ADPKD cysts appears attractive to explain the mysterious link between kidney cysts, aneurysms, liver cysts, and hernias: the law of Laplace. This law allows us to present an alternative, simple hypothesis: the cysts grow because of the increased intratubular pressure and because of the lower wall tension in ADPKD than in a normal kidney.

Kidney cysts, similar to aneurisms, develop over a prolonged time. Therefore, both intratubular pressure and basement membrane compliance are likely near normal values in ADPKD with changes so small to be undetectable. Our point is that once a cyst appears, it keeps growing due to Laplace’s law. Furthermore, the generation of new epithelial cells is needed to accommodate the expansion of the walls of the cysts; without such cell proliferation, the cysts’ wall would rupture. In other terms, cell wall proliferation is a consequence and not the cause of cyst development.

Here, we briefly explain this new hypothesis, show data that support it, and suggest possible therapeutic consequences.

## 2. Laplace’s Law in Kidney Tubules: Hypothesis on ADPKD

**Figure 1:**
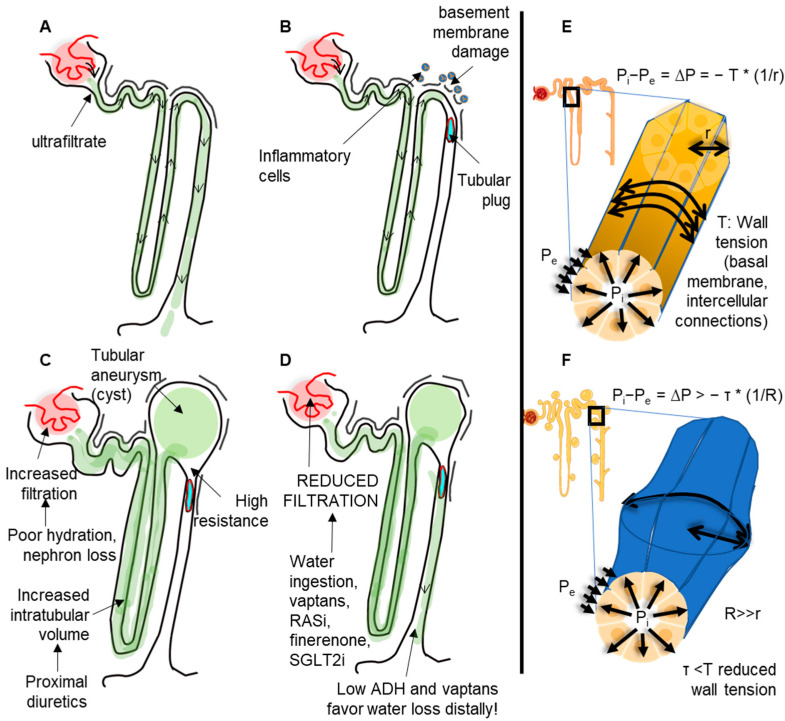
Mechanism of cyst formation from a cellular perspective. (**A**) In normal conditions, ultrafiltration produces a negligeable intratubular pressure that is adequately counterbalanced by the wall tension forces and the external pressure applied onto the tubules. (**B**) In pathologic conditions, such as inflammation or altered molecular composition (e.g., from genetic alterations) of basal membranes, the wall tension can be reduced. The presence of intratubular obstructions may thus favor the increase in intratubular pressure, which is not adequately balanced by the tubular wall tension. (**C**) In conditions of increased ultrafiltration or increased intratubular volume (e.g., by proximal diuretics), the condition in B can induce the formation of tubular aneurisms (cysts). (**D**) Drugs that reduce the glomerular filtration can reduce intratubular pressure and thus slow down aneurism progression (likewise, in arterial aneurisms, the blood pressure must be tightly controlled). (**E**) In normal kidneys, the pressure difference between the inside (Pi) and the outside (Pe) of tubules (∆P) is counterbalanced by the wall tension by a factor depending on the radius. (**F**) Once the tubular aneurism is formed, the aneurism will keep dilating due to Laplace’s law.

In adult dominant polycystic kidney disease (ADPKD), a minimal reduction in wall tension may lead to a dilatation in areas along the nephron. The initial increase in the tubule radius then facilitates the further expansion of the cysts. Since the cell dimension remains constant, the number must increase to guarantee the presence of the cystic wall. Hence, tubular cell proliferation may be at least partially a consequence and not the cause of cyst formation (Figure 1).

Laplace’s law states that, in a tubule, the tangential wall tension must be equal to the inner pressure times the radius of the tubule. In the case of a thin wall, we have:

∆P = −τ ∗ (1/R)

where ∆P is the pressure difference between the inside and outside of a tubular structure, τ is the wall tension, and R is the radius of the tubule. This law is valid for infinitesimally thin walls [32], which is the case in large cysts where the wall thickness is much thinner compared to the radius. In such instances, therefore, Laplace’s law can be considered a good approximation of the system.

This model predicts that aneurysms will form when Po > Pi. The subsequent increase in the radius would require even greater tension, and therefore, the tubule will keep growing. This model explains why cyst growth is absent in ESKD; without filtration, the intratubular pressure is reduced. Hence, the wall tension is sufficient to counterbalance and even win the product (inner pressure × radius).

Since all tubules have organized one close to the other in parallel, Po > Pi can occur (i) in tubules close to the surface of the kidney and (ii) in tubules close to the kidney pelvis.

The large heterogeneity of cysts (clusters of small or large cysts) in ADPKD and in ACKD is compatible with the hypothesis that the reduced tubular wall tension, which is supposed to be homogeneous in all nephrons, has effects on tubular dilation only in the presence of increased internal pressure. Given that the GFR is heterogeneous among nephrons (see, e.g., [33]) with the coexistence of nephrons that filtrate more and others with low GFR, the appearance of dilations is expected to appear randomly and scattered in different nephrons.

According to a 2018 paper by Gilmer et al. [30], the maximum intratubular pressure drop occurs in the thin descending limbs of Henle and the inner medullary collecting ducts. However, the highest resistance to the intratubular flow is within the thin descending limbs where the smallest tubular section can be observed [34]. These authors also predict that proximal diuretics (acetazolamide and SGLT2-inhibitors) give a substantial back pressure in Bowman’s capsule, decreasing the glomerular filtration rate [35].

These authors report that the proximal tubule and Bowman capsule and the collecting duct have higher compliance (0.833 µm/mm Hg) compared to the loop of Henle (0.133 µm/mm Hg) and the distal tubule (considered as inelastic).

The values of intratubular pressure are 8.6 mmHg for the glomerular capsule raising to 15.8 mmHg under water diuretic conditions (increased flow in collecting duct), whereas mannitol and saline diuresis induce a pressure drop of approximately three times in the collecting ducts compared to anti-diuresis. Therefore, in the presence of water diuresis, the authors predict an increase in tubular diameter in a normal kidney maximally at the level of the inner medullary collecting duct (29%) followed by the cortical collecting duct (3.75%) with the remaining tract of the nephron showing minimal dilatation (1.7% proximal tubule, 2% thin descending limb, 0.3% thick ascending limb, 1.25% outer medullary collecting duct). Using these data, it is possible to estimate the wall tension along the nephron (Table 1).

Notably, the wall tension is smaller in the collecting duct where, in standard conditions, the intratubular pressure is the lowest along the nephron.

These data can be compared with the occurrence of cysts in ADPKD along the nephron, as reported in Table 2.

Indeed, in ADPKD, cysts can form both in the cortex and medulla [36]. Although all nephron segments may be affected, the greatest probability of cyst formation occurs in the distal nephron and in the collecting duct (glomerular structures are affected in rare cases [36,37,38,39]), which is close to the medulla, where the external pressure (Po) is reduced. Cysts are often surrounded by smooth muscle cells [40]. Specifically, Grantham reported 7% of cysts in the collecting tubule, 1.8% in the proximal tubule, and 2% glomerular, whereas 84% of cysts were “not typical of normal tubule segments” [29]. In a genetic rodent model of ADPKD (Pkd2 mutation), 40% of the cysts were of collecting tubular origin, 42% distal tubular origin, and 5% proximal tubular origin [41].

Intriguingly, this correlates perfectly with the baseline wall tension of the tubules along the nephron.

The old localization of cysts along the nephron based on Na+ concentration (low Na+ = distal tube; high Na+ = proximal tube) seems to not be appropriate as the histological investigation has shown a continuum of sodium quantity rather than a well-defined categorization [42].

Therefore, Laplace’s law predicts that any initial reduction in wall tension along the nephron would result in an auto-sustained enlargement of the tubules, thus leading to the formation of cysts.

As pointed out by an anonymous reviewer, clusters of cysts of different sizes can be observed, and they might respond differently to vaptans. In an animal model of ADPKD, tubule compression by adjacent cysts (particularly in the collecting duct) might be responsible for tubular atrophy in non-cystic nephrons and atubular glomeruli [29,43].

In ADPKD, cysts of all sizes are present, though only the larger ones can be observed with ultrasound or magnetic resonance (MR). Indeed, with histological techniques, clusters of small cysts below the resolution of US and MR are present in adults, though their growth rate is unknown [44,45].

Despite the large variability in the size of cysts in ADPKD (with large and small cysts co-existing in MR imaging), unfortunately, no data are available about individual cyst growth (only mean size of cysts is available), the degree of cyst heterogeneity over time, and their response to vaptans [33].

Recently, ADPKD cysts have been classified using MR criteria as follows (Toronto updated classification system): (i) class 1 (typical) consisting in bilateral and diffuse distribution of cysts, which contribute to increased kidney volume; (ii) class 2A (atypical) with unilateral, segmental, asymmetric, lopsided, segmental sparring, and mild lopsided; and (iii) class 2B (atypical) with bilateral cysts and kidney atrophy. The presence of atypical class 2 cysts would be more prevalent when no classical ADPKD genes are mutated [33]. No information is available regarding the response of the two classes of cysts to vaptans, though class 2 cysts have a slower progression rate.

The number of cysts in ADPKD correlates with the rate of reduction in eGFR [33]. An analysis of individual cysts over time also shows that cysts increase over years, which correlates with the total kidney volume change and, in turn, with the eGFR [33]. Cysts appear to grow exponentially as a function of time (the growth is linear vs. time on a log scale) [46], in agreement with Laplace’s law. A reanalysis of the data reported in the same manuscript [46] shows that small cysts have a lower growth rate than larger cysts, as expected by Laplace’s law.

In the next section, we will review the extrarenal features of ADPKD, suggesting that they can be explained by a reduced wall tension. This will be validated by the similarity of these extrarenal features and other diseases affecting wall tension in multiple organs. We will then explore the possible molecular mechanisms linking the mutation of the ADPKD genes (PKD1-2) to a reduced cell wall tension in kidney tubules as well as in other organs. Finally, we will review current specific therapies for ADPKD, considering wall tension theory, and propose a series of testable predictions of the theory.

## 3. ADPKD: Major Extrarenal Features

ADPKD is wrongly considered a disease affecting only the kidney. Several tissues and organs are characterized by the presence of this mutation, and some of them develop cysts and/or dilatations. Extrarenal manifestations are extensively reviewed by Bergmann et al. (2018) [37].

These are the major extrarenal manifestations:

- Liver: Approximately 30% of patients with ADPKD develop liver cysts [47], which derive from cholangiocytes [48]. Most patients have their hepatic function unchanged. Pain is a common symptom [49].
- Blood vessels: A higher prevalence of cerebral and aortic aneurysms have been described in ADPKD [50,51]. Furthermore, these patients also form artero-venous fistulas with greater diameter and have a higher chance of developing venous aneurysms [52,53,54].
- Heart: ADPKD has been associated with dilated cardiomyopathy [55]. This has been linked to mitophagy [56] and, similarly, to autophagy occurring in other muscle types [17].
- Pancreas: Patients with ADPKD can show both cysts in pancreatic tissue and, with lower frequency, intraductal papillary mucinous neoplasms (IPMN) [57].
- Male reproductive organs: Cysts have frequently been observed (43% of ADPKD cases) in the rete testis, epididymis, seminal vesicles, and prostate [58].
- Gut: Colonic diverticula occur especially in patients on maintenance dialysis [59]. Extracolonic localization has also been observed [60].
- Abdominal wall: Patients with ADPKD may often present with abdominal wall hernias (45%) [61]. Although the mass effect caused by the enlargement of the kidneys may contribute to this phenotype, a reduced wall tension may also be responsible. Indeed, the placements of catheters, such as in peritoneal dialysis, almost doubles the prevalence of hernias in subjects with ADPKD compared to those without ADPKD [62,63]. Furthermore, nephrectomy, which should lower intra-abdominal pressure, is also associated with incisional hernias [64].
- Ovarian cysts: Single ovarian cysts are frequent in ADPKD [65].
- Lungs: Bronchiectasis has been described [66].
- Brain: No gross brain abnormalities have been described in patients with ADPKD. However, they might suffer from depression [67]. Interestingly, no mild cognitive impairment has been reported in these patients, although it is often present in other forms of CKD [67,68,69,70,71,72,73]. Both PKD1 and PKD2 have large expression in the brain (data from Allen Brain Atlas database and from Human Protein Atlas database). Therefore, their absence may be expected to lead to hyperactivity [74], anxiety [75], and memory disturbances [76].
- Eyes: Subtle modifications of the retina and corneal endothelial cells have been described [77]. Retinal detachment has been described in isolated cases [78], and no systematic statistics are available, though this topic would be relevant for possible therapeutic approaches [79,80].
- Cancer: At present, the role of ADPKD in renal cancer is controversial [31].

The analysis of the extrarenal features of ADPKD offers this scenario: most of the walled organs and tubular structures have an enhanced risk of undergoing aneurismatic alterations. Compact organs without tubular structures, such as the brain, or organs protected in bone cavities, such as the eyes and the brain itself, do not show macroscopic abnormalities.

It should be noted that there is some similarity between the ADPKD extrarenal phenotype and a disease caused by alteration in elastic fibers. Indeed, in Marfan syndrome, a defect in the gene codifying Fibrillin 1 is associated with hepatic and renal cysts as well as arterial aneurysms [81,82]. Fibrillin 1 is a protein essential for the genesis of elastic fibers, and its derangement may disrupt the walls’ response to the hydrostatic intratubular pressure in the kidneys.

## 4. Putative Molecular Links between Polycystin Proteins and Tubule Wall Tension

The mutation of four different genes can give rise to the same ADPKD phenotype: PKD1 and PKD2 (polycystin genes), GANAB (Glucosidase II Alpha Subunit), and DNAJB11 (DnaJ Heat Shock Protein Family (Hsp40) Member B11).

PKD1 encodes for a small membrane-bound protein that interacts with cell junctional complexes [83]. Indeed, it colocalizes with desmosomes [84], a cellular structure devoted to cell–cell interaction, which ensures stable tissue tension. It also interacts with several intermediate-filament structural proteins, such as vimentin, cytokeratins, and desmin [83]. It also interacts with polycystin2 (PKD2 gene), which is, however, an ion channel.

PKD2 mutation leads to a syndrome that looks indistinguishable from PKD1 but has a milder evolution. PKD2 is a membrane cation (Ca+2, K+) channel.

The molecular interactome of the PKD1 and PKD2 protein is here reviewed based on three publicly available databases: STRING (https://string-db.org/, 1 May 2023), IntAct (https://www.ebi.ac.uk/intact/, accessed on 1 May 2023), and the human reference protein interactome mapping project HuRI (http://www.interactome-atlas.org/search, 1 May 2023). All databases report the interaction of PKD1 and PKD2.

PKD1 is an interactor of PKD2 (a cation channel) but further interacts with E3 ubiquitin-protein ligase SIAH1 and nephrocystin-1 NPHP1, the latter being responsible for interaction with the cell matrix adhesions. The STRING database also enlists an interaction with PTK2, which regulates cellular adhesion; vinculin (VCL), which binds to actin filaments in the cytoskeleton and is important for maintaining cell shape; cadherin-1 (CDH1), an adhesion protein; catenin beta-1 (CTNNB1), which also regulates cell adhesion; and tuberin (TSC2), which also regulates microtubule-mediated protein transport.

PKD2 interacts with the keratin-associated proteins (KRTAP, KRT40) responsible for extracellular filaments; the heart proteins troponin and MDFI, which are essential for heart contraction; calcium channels (TRPC1); and other proteins (PLSCR1, MAGEA8, HSF2BP). The STRING database also enlists the same interactors of PKD1.

**Figure 2:**
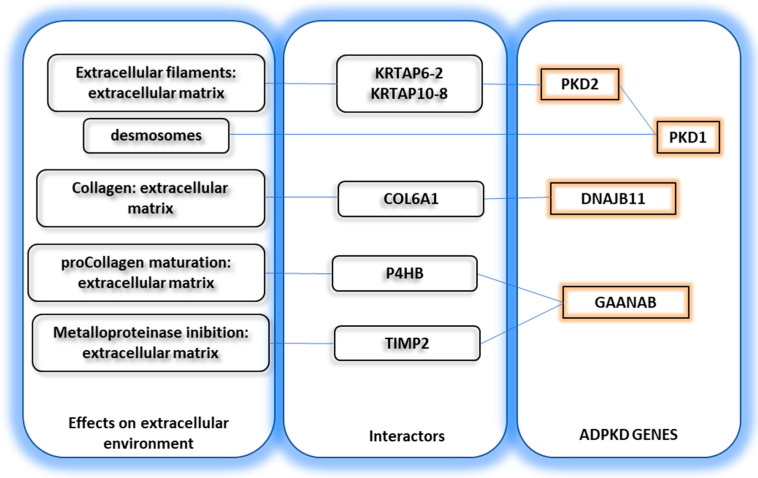
Extracellular matrix proteins interacting with ADPKD genes.

DNAJB11 has several interactors. Among these, COL6A1 is implicated in the formation of collagen type 6. GANAB interacts with P4HB, which is involved in pro-collagen maturation, and with TIMP2, which is involved in metalloproteinase inhibition and, therefore, extracellular matrix remodeling (See Figure 2). Based on the brain distribution of the four ADPKD genes, it would be expected that patients with PKD1/PKD2 deletion (which are poorly expressed in the brain) might show some anxiety [75], whereas those with GANAB/DNAJB11 mutation (highly expressed in the brain) might have hyperactivity or memory loss [76,85].

In line with the hypothetical interaction between the ADPKD genes and extracellular matrix, early studies showed a laminated basement membrane in ADPKD kidneys [8] and altered extracellular matrix gene expression [8].

One theoretical problem addressed by the molecular studies on ADPKD is why cystogenesis is focal. Notwithstanding the presence of mutations in all kidney nephrons, less than 10% of the tubules will develop cysts. Furthermore, within the same affected nephron, only some segments will undergo cystic dilation. This phenomenon has been explained with the “second-hit” hypothesis, suggesting that polycystin may undergo random mutations/DNA damage [86]. Cystogenesis may occur when functional polycystin falls below a critical threshold (through mechanisms reported below) [87,88].

This theory just moves the problem towards other, unknown, random DNA alterations occurring in all subjects and with consequences only if PKD1 or PKD2 are mutated. In other terms, it tries to explain the random spatial effects of PKD mutations using another random variable, DNA mutation.

A significant problem of this approach is that conditions with increased chances of DNA mutation (such as exposure to chemotherapy, mutagens, radiation) should increase cystogenesis, but this has never been observed.

Conversely, the random variation in intratubular pressure/basal membrane tension is sufficient to explain the random appearance of the phenomenon in our theory.

## 5. Acquired Cystic Kidney Disease (ACKD): A Unified View

Isolated simple cysts of the kidney are extremely prevalent in the general population, probably 17–27% [89]. Their prevalence increases with age up to 30–50% in the seventh decade of life [89]. In the case of chronic kidney disease (CKD), the prevalence of multiple cysts is even greater, occurring in up to 90% percent of people on dialysis. In a study on 561 transplanted patients, the native kidneys showed more than three cysts in 23% of cases [90].

Simple cysts show an increased prevalence in cases of hyperparathyroidism (34% vs. 16% in normal patients) [90], correlating, as expected, with lower tubular maximal phosphate reabsorption induced by high PTH.

The prevalence of simple renal cysts increases non-linearly with age, almost doubling every decade of age [33] with a higher prevalence in males than females [33]. Simple renal cysts are associated with hypertension, and the higher the number and size of the cysts, the greater the risk of hypertension [91]. The number of cysts is also inversely correlated with the eGFR and albuminuria [92]; cyst size is also dependent on diabetes mellitus [33]. ACKD does not correlate with a specific etiology (CAKUT, stones, glomerulonephritis, solitary kidney) and is highly frequent in patients on hemodialysis [33].

Indeed, there is some association between renal cysts and diabetes, whereas these are more common in the presence of metabolic syndrome. In a logistic regression analysis, male gender, age, cerebrovascular disease, and increased creatinine are linked to cysts, whereas insulin therapy and diabetic foot are protective [92]. However, the data are not in agreement among studies. A separate study on 436 patients and 436 controls found an association between cysts and hypertension but not with low kidney filtration [92].

The proposed pathogenesis of simple cysts are (1) weakening of the tubular basement membrane and obstruction, similar to the theory proposed above for ADPKD with the formation of diverticula [92], and (2) nephron hypertrophy prompted by renal ischemia.

Seminal experiments suggest that an increase in transepithelial hydrostatic pressure does not explain cyst formation because intracystic pressure is not high enough [8].

Several animal models have been developed to study simple cysts: treatment with diphenylthiazole or nordihydroguaiaretic acid, the genetic C57Bl/6J [cpk/cpk] mouse strain, and the CFWw mouse strain can develop renal cysts. A study in 1987 based on micropipetting suction of the wall of tubules failed to demonstrate altered viscoelastic properties of the tubules in these models [8]. However, a review of the data reported in this study showed an increased deformability of the normal tubules from all animal models compared to the controls (differences which were significant for the nordihydroguaiaretic acid model). Furthermore, independent evidence suggests a remodeling of the basement membrane in one of these models [8]. Finally, the observation of similar deformability of the basement membrane in cysts and normal tubules strengthens our hypothesis that cysts are destined to enlarge because the larger radius of cysts would require greater wall tension.

Recent data further strengthen the alteration of the basement membrane in cysts [8].

Two additional mechanisms should be considered in acquired ACKD and ADPKD: (i) cystic dilation of the tubules upstream of atrophic tubules served by a normally functioning glomerulus and (ii) compression and atrophy of normal nephrons compressed by adjacent cysts. The second mechanism applies particularly to ADPKD where the number of cysts is so large that they compress the non-aneurismatic tubules, causing the presence of “atubular glomeruli” [43]. This mechanism is reported to be comparable to that in ureteral obstruction [43]. Atubular glomeruli represent a puzzling event: they have normal capillaries and Bowman’s capsule, therefore, they are filtrating; however, how the ultrafiltrate is reabsorbed without a tubule is unclear.

## 6. Conclusions and Perspectives

Current theories on cystogenesis in ADPKD have difficulties in explaining the systemic phenotype of the disease and the occurrence of cysts in ACKD. A unitary hypothesis can be proposed based on a reduced wall tension in several structures, which might be linked to the genetic alteration underpinning the disease.

This hypothesis should be considered when designing the treatment of single and multiple kidney cysts. Theoretically, the reduction in intratubular pressure should reduce cyst growth rate. Therefore, drugs that reduce the glomerular filtration rate, such as the rennin–angiotensin inhibitors, would be helpful in this regard. Conversely, drugs that increase intratubular pressure, such as diuretics, might increase cyst growth rate. Vaptans and high water intake are an exception because they increase intratubular pressure only in the distal tubule where the vasopressin receptor is expressed and actually reduce the glomerular filtration rate [8,35], therefore reducing intratubular pressure in the other nephron segments.

## Figures and Tables

**Table 1:** Theoretical wall tension values of different parts of the nephron. Radius and pressures are derived from the 2018 paper by Gilmer et al. Wall tension is calculated as H = P ∗ (r/(2 ∗ T).

	Diameter (µm)	Intratubular Pressure (mmHg)	Wall Tension (τ)
Proximal tubule	23	8.1	0.054
Loop of Henle	15	4	0.027
Distal convoluted tubule	40	4	0.027
Collecting duct	24	2	0.013

**Table 2:** Frequency of occurrence of cysts along the nephron in ADPKD.

Tubular Tract	% of Occurrence
Proximal tubule	1.8–5%
Loop of Henle	Not available
Distal convoluted tubule	42%
Collecting duct	7–40%

## Data Availability

Not applicable.
